# The impact of E-health literacy on patient loyalty in county-level public hospitals: the mediating role of value co-creation and the moderating role of perceived social support

**DOI:** 10.3389/fpubh.2026.1940333

**Published:** 2026-09-09

**Authors:** Mengjiao Yang, Chengxin Fan, Bei Lu, Yifan Wang, Zhongming Chen, Wenqiang Yin

**Affiliations:** 1 School of Management, Shandong Second Medical University, Weifang, Shandong, China; 2 School of Public Health, Shandong Second Medical University, Weifang, Shandong, China; 3 Liuzhou People's Hospital Affiliated to Guangxi Medical University, Liuzhou, Guangxi, China

**Keywords:** E-health literacy, inpatient, patient loyalty, perceived social support, value co-creation

## Abstract

**Objectives:**

This study explores the mechanisms by which E-health literacy among inpatients in county-level public hospitals influences patient loyalty. It further examines the mediating role of value co-creation and the moderating effect of perceived social support, seeking to provide empirical evidence for the digital transformation of county hospitals and patient relationship management.

**Methods:**

A stratified random sampling method was employed to survey 784 inpatients in county-level public hospitals in Shandong Province. E-health literacy, value co-creation, perceived social support, and patient loyalty were assessed using a structured questionnaire. Correlation analysis, regression analysis, and moderated mediation models were applied to examine both the direct and indirect effects of E-health literacy on patient loyalty.

**Results:**

E-health literacy was positively associated with patient loyalty, and value co-creation mediated this relationship. Furthermore, perceived social support was found to moderate the relationship between E-health literacy and patient loyalty, suggesting that elevated perceived social support weakened the positive association between E-health literacy and patient loyalty.

**Conclusion:**

E-health literacy appears to be an important factor associated with inpatient loyalty in county-level public hospitals. Value co-creation and perceived social support play important mediating and moderating roles, respectively, within this process. By enhancing patients’ E-health literacy, promoting value co-creation behaviors, and leveraging social support resources, it is possible to foster sustained improvements in patient loyalty and advance the quality of smart healthcare services in county-level public hospitals.

## 1 Introduction

### 1.1 Research background

With the ongoing global information technology revolution and the continual evolution of health management concepts, digital healthcare, medical informatization, and the development of “smart hospitals” have emerged as pivotal forces driving the transformation and upgrading of healthcare systems (1, 2). The World Health Organization has explicitly set goals to promote universal health coverage and health-related sustainable development through digital technology, signifying that digital health has become a central item on the global public health governance agenda (3). As of June 2025, the 56th Statistical Report on China’s Internet Development, published by the China Internet Network Information Center (CNNIC), indicates that the number of internet users in China has reached 1.123 billion, showing an increase of 14.36 million compared with December 2024. The internet penetration rate stands at 79.7%, and there are 393 million online medical service users, accounting for 35.0% of all internet users (4). Nationwide cross-sectional research on Chinese residents further confirms that over 42.3% of respondents have searched for health information online, and nearly 33% have relied on online health resources for preliminary self-assessment of physical discomfort (5). In addition, a survey conducted in the United States reveals that 59% of respondents have experience in searching for health information online, and 35% have used online health information for self-diagnosis of health conditions (6). Thus, E-health literacy has gradually become a critical factor in shaping doctor-patient relationships, improving healthcare service quality, and enhancing patient outcomes (7).

County-level public hospitals undertake grassroots medical services but face obvious resource constraints, including insufficient digital infrastructure and limited channels for residents to obtain health information compared with urban large hospitals (8). Moreover, inpatients usually suffer from complex illnesses and long treatment cycles, and they have higher demands for continuous and coordinated digital medical services (9). Existing literature confirms that improving inpatients’ E-health literacy within resource-limited county medical environments can optimize doctor-patient communication, raise service efficiency (10), improve patient experience (11), and ultimately cultivate patient loyalty (12).

Although previous studies have examined the influence of E-health literacy on various aspects such as patients’ medical decision-making, health management, and healthcare utilization behaviors (13), there remains a lack of in-depth and systematic empirical research on how E-health literacy specifically affects patient loyalty within the digital healthcare environment, particularly in the context of county-level public hospitals. The underlying pathways and mechanisms through which this relationship operates remain underexplored. To address these research gaps, this study introduces value co-creation and perceived social support as core variables. Relevant theoretical reasoning and hypothesis development are elaborated in the following subsection.

### 1.2 Theoretical background and research hypotheses

#### 1.2.1 Theoretical background

E-health literacy (E-health) was first conceptualized by Norman and Skinner as a multidimensional construct integrating six core competencies: traditional literacy, health literacy, information literacy, scientific literacy, media literacy, and computer literacy. It emphasizes individuals’ comprehensive ability to access, understand, evaluate, and apply health information in digital health environments to address health-related problems (14). With the increasing availability of e-health applications, telemedicine, and intelligent health management services, patients’ demand for and reliance on digital health information have grown substantially. A substantial body of empirical research has confirmed that E-health literacy positively influences patients’ health behaviors, treatment adherence, healthcare experiences, and satisfaction (15, 16).

Patient loyalty (Loyalty) serves as a core indicator for assessing the quality of healthcare services and the sustainable development of medical institutions. It is manifested not only through explicit behaviors such as repeat visits, proactive referrals, and voluntary recommendations, but also indirectly influences hospital brand building, disciplinary development, and resource integration capacity (17), garnering increasing attention in recent years. Patient loyalty is defined as the willingness to choose the same institution for future healthcare needs or to recommend it to family and friends (18). Existing research shows that loyal patients can improve health outcomes by enhancing adherence to treatment regimens (19, 20). Moreover, patient loyalty has been found to generate stable demand for medical services through increased referrals and positive word-of-mouth, reinforce hospital reputation, and foster positive doctor-patient relationships (17, 21), thereby promoting the market competitiveness of medical institutions. In the context of rapid digital health development, E-health literacy has emerged as a crucial antecedent in shaping patient loyalty. As a core competency that enables patients to comprehend, evaluate, and apply health information in a digital environment, E-health literacy not only enhances patients’ sense of information control and self-efficacy (14), but also encourages more active and in-depth participation in medical decision-making and health management. This, in turn, strengthens patients’ trust, identification, and dependence on healthcare institutions (22), ultimately facilitating the stable formation of loyal behaviors (12).

The theory of value co-creation (CValue), originally proposed by Prahalad and Ramaswamy, emphasizes the role of enterprises and users as equal participants who, through continuous dialogue and interaction, jointly create personalized experiences (23). Within healthcare research, value co-creation is further differentiated into in-hospital and out-of-hospital value co-creation, with the former referring to patients’ active engagement in clinical encounters such as shared decision-making and treatment planning, and the latter referring to health information seeking and self-management activities beyond the hospital setting (24). In modern healthcare systems, patients with high levels of E-health literacy are able to more effectively utilize digital platforms and intelligent medical tools to actively participate in the collection and analysis of health data, articulate their health needs and treatment preferences, and engage with healthcare teams in jointly making decisions and developing personalized treatment plans (22, 25). As a result, patients shift from being passive recipients of medical care to becoming active co-creators (24). This transformation not only enhances the effectiveness of medical services and patient satisfaction, but also lays a solid foundation for continuous innovation and development of healthcare systems. In addition, the positive effect of value co-creation on customer loyalty is well recognized in the service literature (26). Based on this reasoning, it is plausible that value co-creation may explain the mechanism by which E-health literacy translates into patient loyalty. Accordingly, we propose that value co-creation mediates the relationship between E-health literacy and patient loyalty.

Perceived social support (PSU), as a key psychological resource, can mitigate psychological stress responses, reduce mental tension, and enhance social adaptation through an individual’s connections with others (27, 28). PSU emphasizes an individual’s subjective perceptions and experiences of social support (29). Research has shown that elevated PSU can markedly increase patients’ trust and satisfaction with healthcare services, reinforce their dependence on and sense of belonging to medical institutions, and thereby foster loyal patient behavior and enhance their willingness to engage in long-term healthcare services (30). However, drawing on Resource Substitution Theory (31), perceived social support, as an external psychological resource, may interact with E-health literacy in shaping patient loyalty. Patients with sufficient emotional and informational support from family and acquaintances tend to rely less on their own digital health capabilities when making health-related judgments. Consequently, the positive effect of E-health literacy on patient loyalty is expected to be weakened among patients with higher levels of perceived social support. Thus, we propose that perceived social support negatively moderates the positive relationship between E-health literacy and patient loyalty.

#### 1.2.2 Research hypotheses

**Figure 1:**
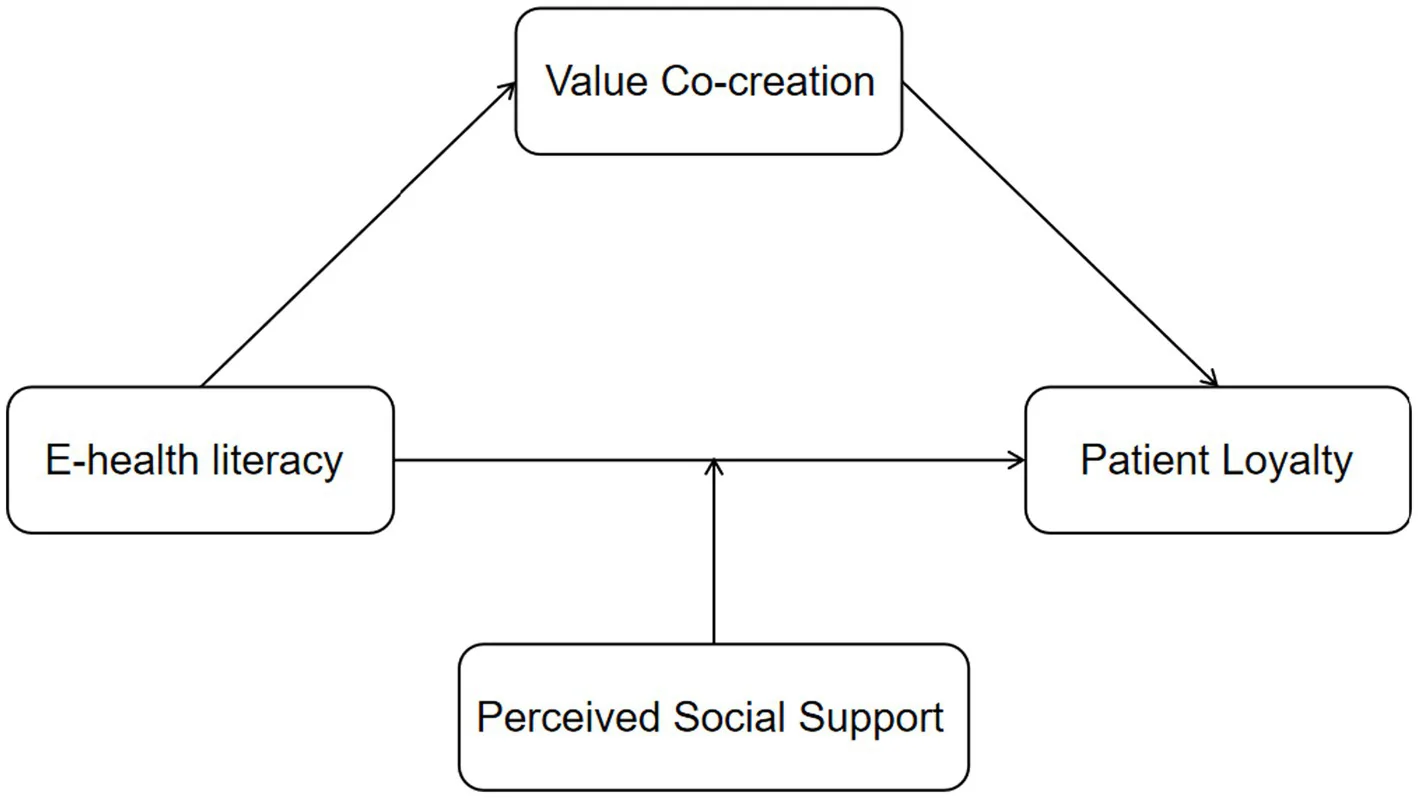
The model of e-health literacy’s effect on patient loyalty.

Based on the theoretical review above, the following conceptual model is constructed (see Figure 1), and the following hypotheses are proposed:

*H1*: E-health literacy is positively associated with patient loyalty.

*H2*: Value co-creation mediates the relationship between E-health literacy and patient loyalty.

*H3*: Perceived social support negatively moderates the positive relationship between E-health literacy and patient loyalty.

## 2 Methods

### 2.1 Participants

A stratified random sampling method was employed in this study. Sixteen municipalities in Shandong Province were included, from each of which three counties representing high, medium, and low economic-development levels were selected, resulting in 48 county-level sampling units. Within these selected counties, 71 secondary or higher-level public hospitals were enrolled. Approximately 10 to 15 inpatients were randomly recruited from each participating hospital. In total, 830 questionnaires were distributed, and 784 valid responses were finally obtained, corresponding to an effective response rate of 94.46%. Exclusion criteria included: patients with severe mental disorders or cognitive impairments at the time of the survey that prevented effective communication or cooperation; individuals unable or unwilling to use electronic devices to access the internet for any reason.

### 2.2 Research instruments and scales

#### 2.2.1 E-health literacy

E-health literacy was measured using the Chinese version of the eHealth Literacy Scale (eHEALS), originally developed by Norman et al. and adapted by Guo Shuaijun (32, 33). The scale consists of 8 items across three dimensions: the application of online health information and services, evaluation skills, and decision-making abilities. Each item is rated on a 5-point Likert scale, where 1 indicates “strongly disagree,” 2 “disagree,” 3 “neutral,” 4 “agree,” and 5 “strongly agree.” The total score, ranging from 8 to 40, is the sum of all item scores, with higher scores representing higher levels of E-health literacy. In this study, the Cronbach’s alpha coefficient for the scale was 0.954, indicating excellent internal consistency.

#### 2.2.2 Patient loyalty

Patient loyalty was assessed with reference to the measurement items developed by Katja et al. and the research by Li et al. (17, 34). The scale included five items: willingness to choose the institution for first consultation, willingness to engage in positive word-of-mouth, willingness to recommend, willingness to revisit, and resistance to alternative providers. Each item was rated on a 5-point Likert scale, with 1 indicating “strongly disagree,” 2 “disagree,” 3 “neutral,” 4 “agree,” and 5 “strongly agree.” The total score is the sum of all item scores, with higher scores indicating higher patient loyalty. In this study, the Cronbach’s alpha coefficient for the scale was 0.893, demonstrating good internal consistency.

#### 2.2.3 Value co-creation

This study drew on the research of McColl-Kennedy et al. and Peng et al. to measure patient value co-creation behavior across two dimensions: in-hospital value co-creation and out-of-hospital value co-creation behaviors (24, 35). The scale employed a 5-point Likert rating, where 1 indicates “strongly disagree,” 2 “disagree,” 3 “neutral,” 4 “agree,” and 5 “strongly agree.” The total score is the sum of all item scores, with higher scores indicating greater patient engagement in value co-creation. In this study, the Cronbach’s alpha coefficient for the scale was 0.926, indicating excellent internal consistency.

#### 2.2.4 Perceived social support

Perceived social support was measured using the Perceived Social Support Scale developed by Blumenthal et al. and adapted into Chinese by Jiang (36, 37). The scale consists of 12 items and evaluates perceived support from three dimensions: others (4 items), family (4 items), and friends (4 items). Each item is rated on a 7-point Likert scale, where 1 indicates “strongly disagree” and 7 indicates “strongly agree.” The score for each dimension is the sum of its item scores. In this study, the Cronbach’s alpha coefficient of the scale was 0.969, indicating excellent internal consistency.

### 2.3 Statistical analysis

R language, SPSS 26.0, and AMOS 26.0 were utilized for statistical analysis in this study. First, Harman’s single-factor test was conducted to assess potential common method bias. Second, descriptive statistics were used to report the mean and standard deviation of each variable. Then, intraclass correlation coefficients (ICCs) were estimated using empty random-intercept models to quantify the potential clustering effect at the hospital level. The Shapiro–Wilk test was used to assess the normality of the continuous scale scores. Given the relatively large sample size and the fact that the bias-corrected Bootstrap method does not rely on the normality assumption, parametric methods were adopted for the primary analyses. Nonparametric sensitivity analyses, including Spearman’s rank-order correlations and Mann–Whitney U/Kruskal-Wallis tests, were additionally conducted to assess the robustness of the findings. Pearson correlation coefficients were then calculated to examine the relationships and significance levels among the variables. In addition, independent samples t-tests and one-way analysis of variance (ANOVA) were employed to examine differences in the core study variables across demographic characteristics. Confirmatory factor analysis (CFA) was performed using AMOS 26.0 to evaluate the construct validity of the measurement model. Finally, Model 4 and Model 5 from the PROCESS 4.1 macro were employed to test the mediating effect of value co-creation and the moderating effect of perceived social support, with the bias-corrected nonparametric percentile Bootstrap method used for effect inference to ensure the robustness and accuracy of the results.

## 3 Results

### 3.1 Data quality assessment

Based on the method for testing common method bias proposed by Podsakoff et al., Harman’s single-factor test was used to assess whether serious common method bias existed in this study (38). The results of the unrotated factor analysis showed that five factors with eigenvalues greater than one were extracted from all items, and the first factor accounted for 43.616% of the total variance, which is below the critical threshold of 50%. This indicates that there is no serious common method bias in the study.

The Shapiro–Wilk test revealed significant deviations from normality for the continuous scale scores (*p* < 0.05). Nonparametric sensitivity analyses, including Spearman’s rank-order correlations, Mann–Whitney U and Kruskal-Wallis tests, produced results consistent with the primary parametric analyses with respect to statistical significance and effect direction, suggesting that the observed deviations from normality are unlikely to substantially compromise the validity of the statistical analyses.

The ICCs for the four core variables were 0.060 for E-health literacy, 0.158 for value co-creation, 0.139 for patient loyalty, and 0.069 for perceived social support, indicating that 6.0 to 15.8% of the total variance was attributable to between-hospital differences, with the remainder at the individual level. Although some clustering was observed, our study focuses on individual-level psychological mechanisms, as all variables and hypothesized relationships are conceptualized at the individual level. Accordingly, single-level analytical approaches using the PROCESS macro were adopted for hypothesis testing.

In addition, the variance inflation factor (VIF) test showed that all VIF values for the study variables were less than 5, and tolerance values were greater than 0.2, indicating that there were no issues of multicollinearity.

### 3.2 Confirmatory factor analysis

The results of the confirmatory factor analysis supported the construct validity of the measurement model. The model showed an acceptable fit to the data, with CFI = 0.895, TLI = 0.887, RMSEA = 0.076, and SRMR = 0.036. Standardized factor loadings were above the recommended threshold, while CR values exceeded 0.70 and AVE values exceeded 0.50 for all constructs, supporting satisfactory internal consistency and convergent validity.

### 3.3 Demographic characteristics of the participants

Table 1 presents the basic characteristics of the 784 hospitalized patients. Among them, 333 were male (42.47%) and 451 were female (57.53%). A total of 378 patients (48.21%) had long-term residence in urban areas, while 326 (41.58%) resided in rural areas. The majority were married, totaling 683 individuals (87.12%). The most common educational level was junior high school, with 253 patients (32.27%). The predominant monthly income group was 3,001–5,000 yuan (28.70%). For more details, see Table 1.

**Table 1:** Basic characteristics of the study participants (*N* = 784).

Variable	*N*	%
Gender		
Male	333	42.47
Female	451	57.53
Age (years)		
≤ 30	120	15.31
31–44	232	29.59
45–59	203	25.89
> 60	229	29.21
Residence		
Urban	378	48.22
Rural	326	41.58
Urban–Rural Fringe	80	10.20
Marital status		
Married	683	87.12
Unmarried/Divorced/Widowed	101	12.88
Educational level		
Primary or below	151	19.26
Junior high	253	32.27
Senior high/vocational	181	23.09
College	102	13.01
Bachelor or above	97	12.37
Monthly income (RMB)		
<1,000	143	18.24
1,001–3,000	210	26.79
3,001–5,000	225	28.70
>5,000	206	26.27

Urban–Rural Fringe refers to village-level areas partially connected to urban public and residential facilities.

### 3.4 Differential testing of demographic characteristics

Table 2 displays the results of the univariate analysis. There were significant differences in CValue and PSU scores among different age groups; specifically, the ≤ 30 and 45–59 age groups had higher PSU scores, while the ≥ 60 age group had lower CValue and PSU scores. In terms of marital status, the married group scored significantly higher on both CValue and PSU compared to the unmarried/divorced/widowed group. Income also showed significant differences in E-health, CValue, and PSU scores, with higher-income groups generally achieving higher scores across these variables. For details, see Table 2.

**Table 2:** Differential testing of demographic characteristics (*N* = 784).

Variable	E-health		CValue		PSU		Loyalty	
	Total scores	*t/F*	Total scores	*t/F*	Total scores	*t/F*	Total scores	*t/F*
Gender								
Male	34.26 ± 6.52	0.307	55.59 ± 6.25	0.591	75.06 ± 11.52	0.725	23.00 ± 3.19	0.464
Female	34.12 ± 6.52		55.31 ± 6.80		74.65 ± 12.72		22.82 ± 3.43	
Age (years)								
≤30	34.96 ± 6.30	2.289	55.91 ± 6.08	3.737**	76.63 ± 10.75	3.407**	22.96 ± 3.59	0.585
31–44	34.63 ± 5.84		56.00 ± 6.80		74.67 ± 12.83		22.66 ± 3.83	
45–59	34.19 ± 6.70		55.85 ± 6.65		76.05 ± 10.71		23.05 ± 2.81	
≥60	33.31 ± 7.04		54.21 ± 7.18		72.94 ± 13.33		22.97 ± 3.06	
Residence								
Urban	34.24 ± 6.20	0.155	55.76 ± 6.43	1.032	75.01 ± 12.06	0.154	22.86 ± 3.57	0.040
Rural	34.21 ± 6.73		55.05 ± 6.39		74.54 ± 12.29		22.93 ± 3.01	
Urban–rural fringe	33.80 ± 7.15		55.41 ± 7.83		75.13 ± 12.84		22.91 ± 3.41	
Educational level								
Primary or below	33.95 ± 7.14	1.804	54.12 ± 7.22	2.542	72.97 ± 14.00	3.775	22.79 ± 3.22	0.511
Junior high	33.75 ± 6.30		55.36 ± 5.59		73.51 ± 12.69		22.98 ± 2.61	
Senior high/vocational	33.93 ± 6.61		56.06 ± 5.97		76.00 ± 9.91		23.08 ± 3.13	
College	34.60 ± 6.73		55.44 ± 8.71		75.80 ± 13.09		22.54 ± 4.43	
Bachelor or above	35.69 ± 5.46		56.46 ± 6.12		77.91 ± 10.11		22.89 ± 4.13	
Marital status								
Married	34.25 ± 6.39	0.790	55.60 ± 6.57	1.970*	75.26 ± 11.60	2.628**	22.93 ± 3.38	0.755
Unmarried/Divorced/Widowed	33.70 ± 7.35		54.23 ± 6.48		71.85 ± 15.56		22.66 ± 2.98	
Monthly income (RMB)								
<1,000	33.24 ± 7.28	3.513**	52.73 ± 7.75	10.866***	70.80 ± 15.17	10.029***	22.56 ± 3.35	0.648
1,001–3,000	33.97 ± 6.16		55.92 ± 6.12		74.48 ± 12.38		22.92 ± 2.90	
3,001–5,000	33.89 ± 6.68		55.60 ± 6.53		74.84 ± 12.19		23.04 ± 3.46	
>5,000	35.36 ± 6.00		56.54 ± 5.65		77.95 ± 8.48		22.95 ± 3.58	

E-health, E-health literacy; CValue, Value Co-Creation; PSU, Perceived social support.

Loyalty, Patient Loyalty; *p < 0.05. **p < 0.01. ***p < 0.001.

### 3.5 Correlation analysis

**Figure 2:**
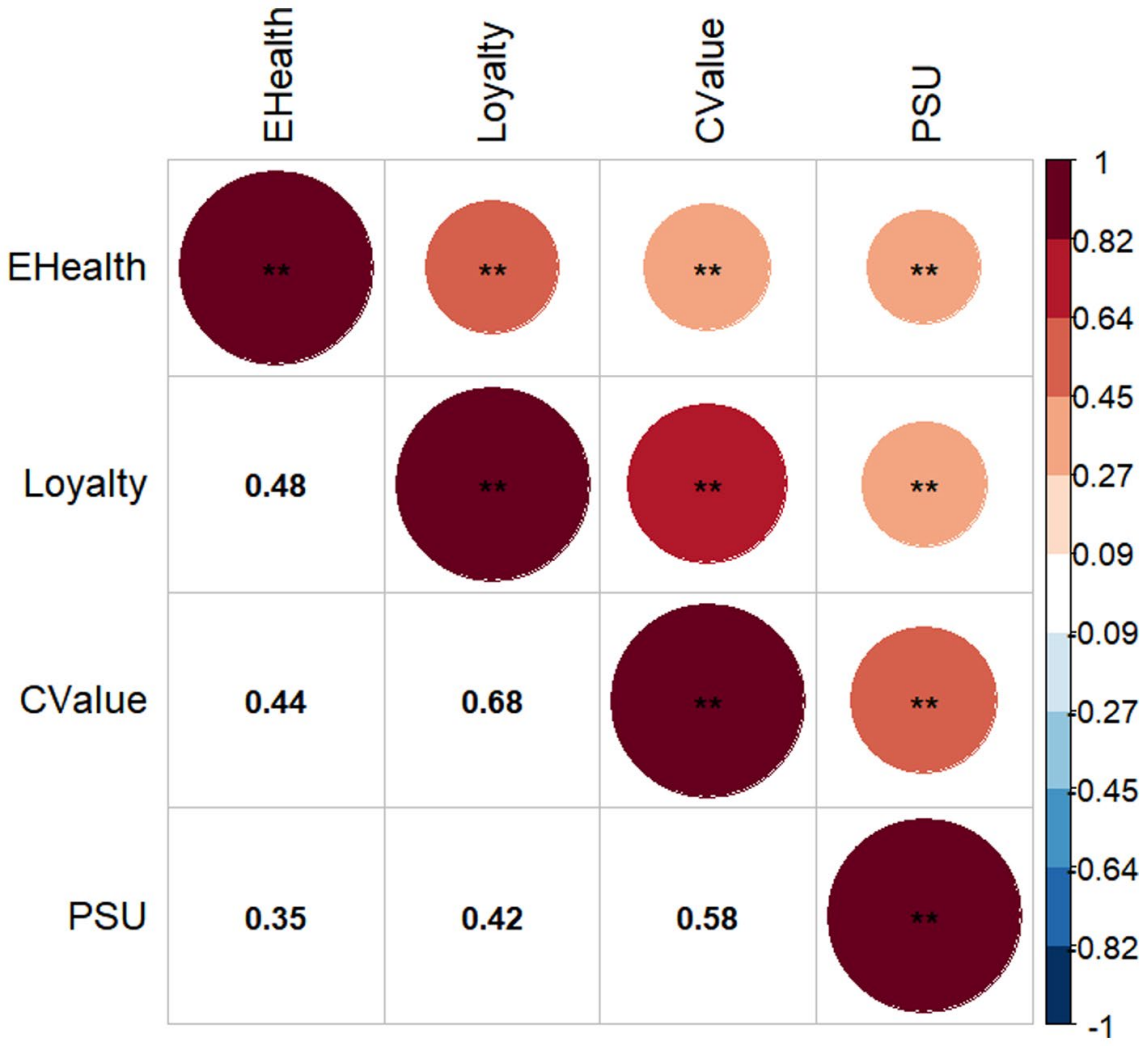
Correlation analysis. E-health, E-health literacy; CValue, Value Co-Creation; PSU, Perceived social support; Loyalty, Patient Loyalty; *p* < 0.01.

Prior to testing the mediating effect, Pearson correlation analysis was conducted to examine the relationships among the variables. The results showed that E-health literacy was positively correlated with patient loyalty (*r* = 0.480, *p* < 0.01), value co-creation (*r* = 0.436, *p* < 0.01), and perceived social support (*r* = 0.351, *p* < 0.01). Value co-creation was positively associated with patient loyalty (*r* = 0.677, *p* < 0.01), and perceived social support was positively associated with patient loyalty (*r* = 0.418, *p* < 0.01). For details, see Figure 2.

### 3.6 Mediation effect analysis

**Figure 3:**
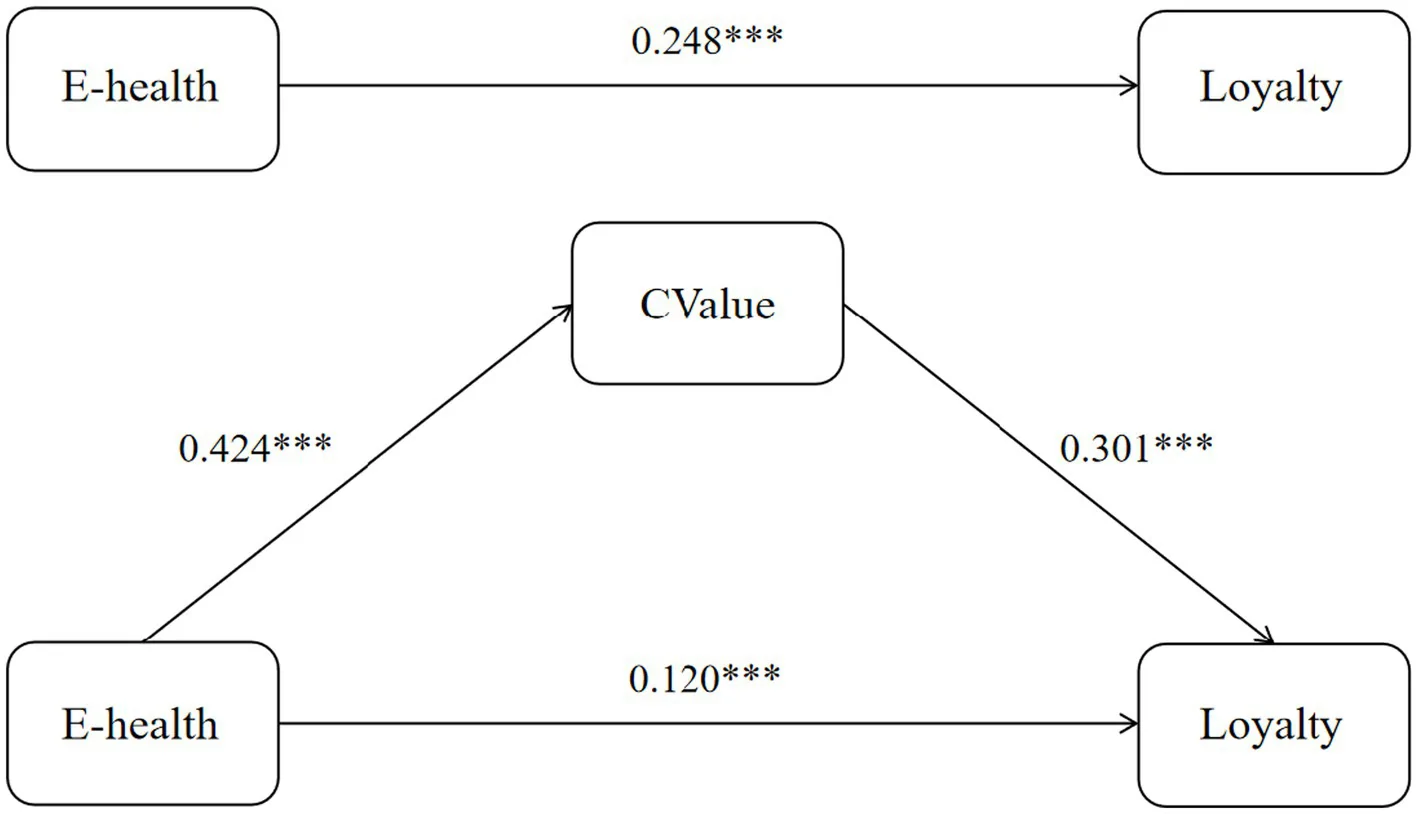
Path coefficients diagram of the mediation model. E-health, E-health literacy; CValue, Value Co-Creation; PSU, Perceived social support; Loyalty, Patient Loyalty; ***p < 0.001.

To explore the mechanism by which E-health literacy enhances patient loyalty, this study further examined the mediating effect of value co-creation between the two variables. The results showed that E-health literacy exhibited a significant positive predictive effect on patient loyalty (*β* = 0.248, *t* = 15.328, *p* < 0.001), supporting Hypothesis H1. After including value co-creation as a mediating variable, the direct effect of E-health literacy on patient loyalty remained significant (*β* = 0.120, *p* < 0.001). Additionally, E-health literacy significantly and positively predicted value co-creation (*β* = 0.424, *p* < 0.001), and value co-creation significantly and positively predicted patient loyalty (*β* = 0.301, *p* < 0.001). The mediating effect was further tested using the bias-corrected nonparametric Bootstrap method. The results indicated that the total effect of E-health literacy on patient loyalty was 0.248 (95% CI [0.216, 0.279]), with a direct effect of 0.120 (95% CI [0.092, 0.148]) and an indirect effect via value co-creation of 0.128 (95% CI [0.086, 0.170]), with all confidence intervals excluding zero. These findings suggest that value co-creation plays a mediating role in the relationship between E-health literacy and patient loyalty, with the mediating effect accounting for 51.61% of the total effect, thus supporting Hypothesis H2. For further details, see Tables 3, 4 and Figure 3.

**Table 3:** Mediation effect test (*N* = 784).

Model	Model 1				Model 2				Model 3			
	*β*	SE	*t*	*p*	*β*	SE	*t*	*p*	*β*	SE	*t*	*p*
Constant	14.213	1.139	12.479	<0.001	41.305	2.288	18.055	<0.001	1.776	1.082	1.642	0.101
Gender	−0.068	0.223	−0.306	0.760	−0.071	0.449	−0.159	0.874	−0.047	0.178	−0.263	0.792
Age	0.171	0.118	1.449	0.148	−0.208	0.237	−0.877	0.381	0.234	0.094	2.483	0.012
Residence	0.030	0.169	0.175	0.861	−0.067	0.340	−0.196	0.845	0.050	0.135	0.369	0.713
Education	−0.067	0.103	−0.646	0.519	−0.014	0.206	−0.070	0.944	−0.062	0.082	−0.757	0.449
Marital Status	−0.096	0.317	−0.303	0.762	−1.132	0.636	−1.779	0.076	0.245	0.253	0.969	0.333
Income	0.039	0.114	0.342	0.732	0.663	0.229	2.900	0.004	−0.161	0.091	−1.762	0.078
E-health	0.248	0.016	15.328	<0.001	0.424	0.033	13.047	<0.001	0.120	0.014	8.451	<0.001
CValue									0.301	0.014	21.14	<0.001
*F*	34.072***				29.129***				102.835***			
R^2^	0.235				0.208				0.515			

E-health, E-health literacy; CValue, Value Co-Creation; PSU, Perceived social support; Loyalty, Patient Loyalty; ***p < 0.001.

**Table 4:** Decomposition of total effect, direct effect, and mediating effect (*N* = 784).

Effect	Effect value	SE	LLCI	ULCI	Effect proportion
Total effect	0.248	0.016	0.216	0.279	100%
Direct effect	0.120	0.014	0.092	0.148	48.39%
Indirect effect	0.128	0.021	0.086	0.170	51.61%

SE, standard error; LLCI, lower limit confidence interval; ULCI, upper limit confidence interval.

### 3.7 Moderation effect analysis

To test the moderated mediation effect, this study constructed two regression equations while controlling for variables such as gender, age, educational level, and residence. As shown in Table 5, after controlling for these variables, the interaction term between E-health literacy and perceived social support had a significant negative effect on patient loyalty (*β* = −0.003, *t* = −3.608, *p* < 0.001, 95% CI [−0.004, −0.001]). This indicates that when perceived social support reaches a certain level, patients are more likely to rely on external support, thereby diminishing the positive impact of E-health literacy on patient loyalty, supporting Hypothesis H3. For details, see Table 5.

**Table 5:** Test of moderation effect (*N* = 784).

Model	Model 1				Model 2			
	*β*	SE	*t*	*p*	*β*	SE	*t*	*p*
Constant	55.779	1.998	27.923	<0.001	6.970	1.238	5.630	<0.001
Gender	−0.071	0.449	−0.159	0.874	−0.049	0.177	−0.278	0.781
Age	−0.208	0.237	−0.877	0.381	0.215	0.094	2.295	0.022
Residence	−0.067	0.340	−0.196	0.845	0.054	0.134	0.399	0.690
Education	−0.015	0.206	−0.070	0.944	−0.045	0.081	−0.557	0.578
Marital Status	−1.132	0.636	−1.779	0.076	0.174	0.252	0.688	0.492
Income	0.663	0.229	2.900	0.004	−0.168	0.091	−1.851	0.065
E-health	0.424	0.033	13.047	<0.001	0.115	0.014	8.021	<0.001
CValue					0.285	0.017	17.161	<0.001
PSU					−0.001	0.009	−0.166	0.868
E-health*PSU					−0.003	0.001	−3.608	<0.001
*F*	29.129***				84.809***			
R^2^	0.208				0.523			

E-health, E-health literacy; CValue, Value Co-Creation; PSU, Perceived social support; Loyalty, Patient Loyalty; ***p < 0.001.

### 3.8 Simple slope analysis

**Figure 4:**
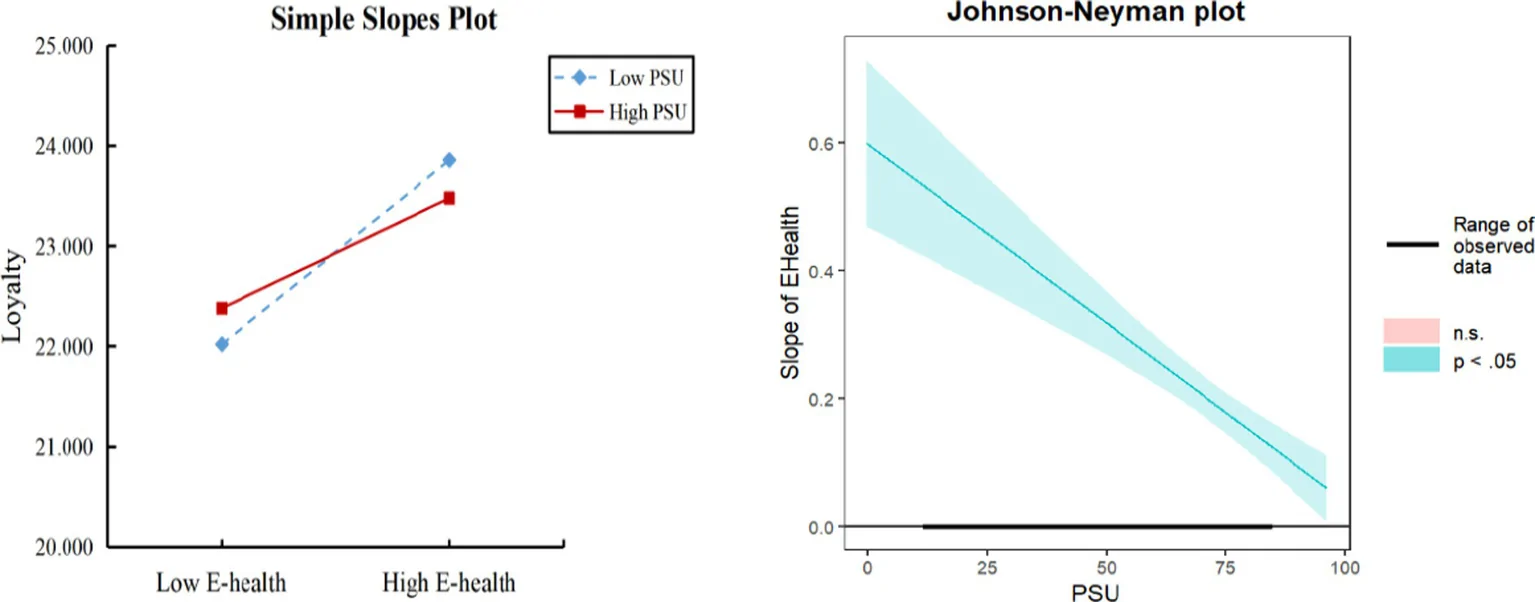
The moderating effect of PSU on the relationship between E-health and loyalty. E-health, E-health literacy; PSU, Perceived social support; Loyalty, Patient Loyalty.

To further explore how the moderating effect manifests across different levels of perceived social support among inpatients, the average score of perceived social support was used as the baseline, with the “maximum” condition (the highest observed value of PSU) defined as high perceived social support, and one standard deviation below the mean defined as low perceived social support. Simple-slope analysis was conducted to estimate conditional direct effects of E-health literacy on patient loyalty at these three levels. Further analysis revealed that under low perceived social support, the conditional direct effect was *β* = 0.149 (95% CI [0.117, 0.181]); in the mean group, *β* = 0.115 (95% CI [0.087, 0.143]); and under high (maximum) perceived social support, the conditional direct effect was *β* = 0.089 (95% CI [0.056, 0.121]). None of the confidence intervals included zero, and the effect size decreased sequentially, indicating that perceived social support significantly moderated the direct relationship between E-health literacy and patient loyalty at all levels. This presents a weakening moderation effect—that is, the higher the level of perceived social support, the weaker the direct pathway by which E-health literacy influences patient loyalty. For details, see Table 6 and Figure 4.

**Table 6:** Conditional direct effects of E-health literacy on patient loyalty at different levels of perceived social support (*N* = 784).

PSU	*β*	SE	*t*	*p*	BootLLCI	BootULCI
M-1SD	0.149	0.016	9.046	<0.001	0.117	0.181
M	0.115	0.014	8.021	<0.001	0.087	0.143
maximum	0.089	0.017	5.384	<0.001	0.056	0.121

SE, standard error; LLCI, lower limit confidence interval; ULCI, upper limit confidence interval.

**Figure 5:**
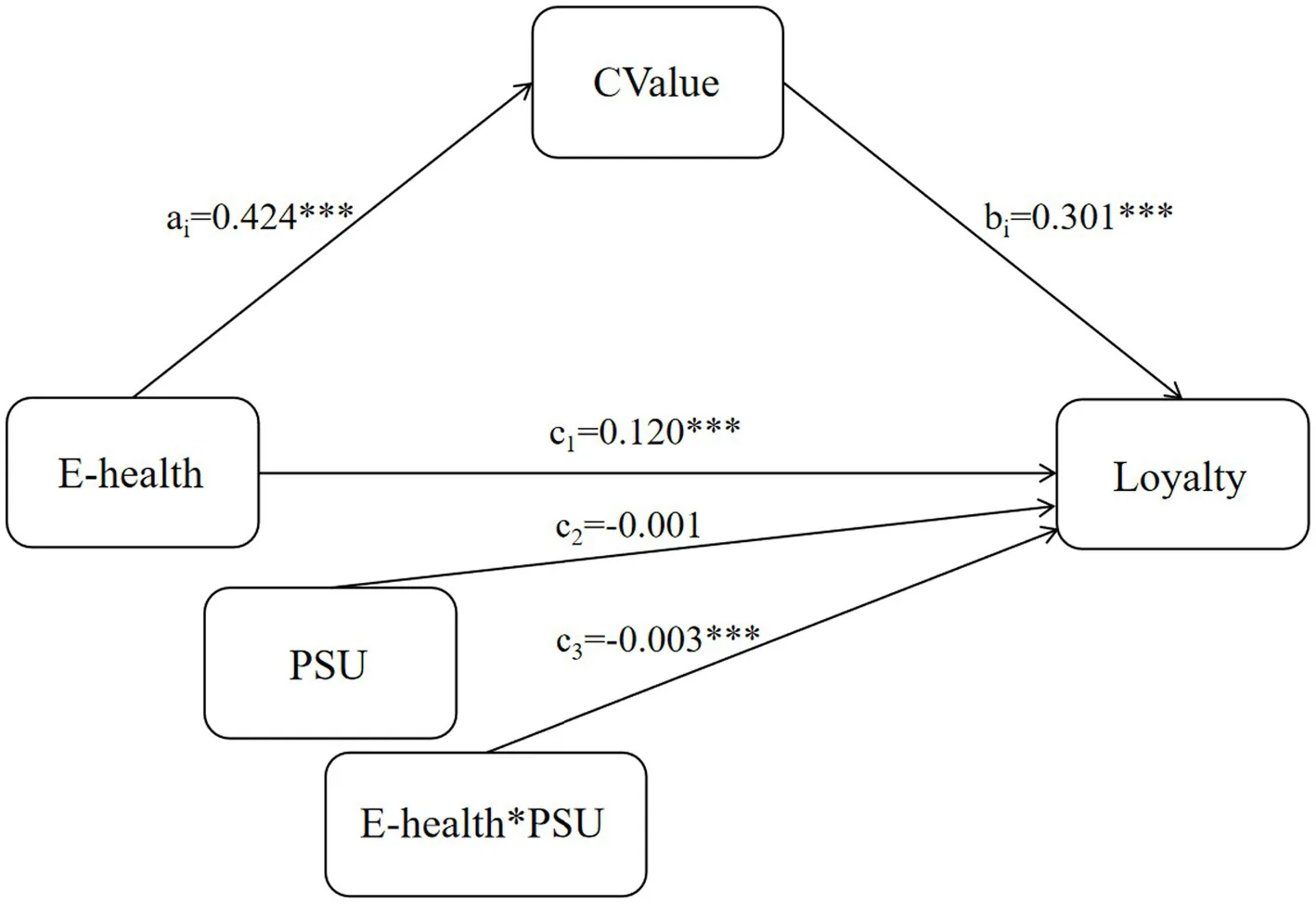
Path coefficient diagram of the moderated mediation model. E-health, E-health literacy; CValue, Value Co-Creation; PSU, Perceived social support; Loyalty, Patient Loyalty; ***p < 0.001.

In summary, the study found that E-health literacy not only directly and positively influences patient loyalty (*β* = 0.248, *p* < 0.001), but also exerts an indirect positive effect on patient loyalty through value co-creation (*β* = 0.120, *p* < 0.001). Additionally, perceived social support demonstrated a significant moderating effect on the direct pathway in the model (*β* = −0.003, *p* < 0.001). For details, see Figure 5.

## 4 Discussion

This study focused on inpatients in county-level public hospitals and systematically explored the mechanism by which E-health literacy influences patient loyalty by constructing a moderated mediation model. The findings reveal that E-health literacy not only shows a significant direct positive association with patient loyalty but also enhances patient loyalty indirectly by promoting value co-creation behaviors. Furthermore, perceived social support demonstrates a significant moderating effect in the process by which E-health literacy influences patient loyalty.

This study identified significant demographic differences in the levels of the core study variables. Consistent with previous research (10), patients aged 60 years and above exhibited lower levels of value co-creation and perceived social support compared to younger patients, highlighting the vulnerability of older adults in the digital health era. Married patients scored significantly higher on both value co-creation and perceived social support than their unmarried, divorced, or widowed counterparts, corroborating the established beneficial role of social support in shaping patients’ healthcare perceptions (27). Higher income levels were similarly associated with greater E-health literacy, reinforcing prior evidence from the Chinese population linking socioeconomic status to health-related digital engagement (15). Of note, patient loyalty demonstrated relatively narrow score variance in our sample. This restricted range might lower statistical power, and could in part account for the absence of significant demographic differences for patient loyalty shown in Table 2.

The relationship between E-health literacy and patient loyalty among inpatients in county-level public hospitals was investigated. The results showed that E-health literacy positively predicts patient loyalty. Specifically, patients with high levels of E-health literacy are more proactive in participating in the medical process, demonstrate greater autonomy over healthcare information, and this not only improves the scientific basis of patient decision-making, but also increases recognition and trust in medical services (11, 15, 39), thereby fostering more stable and sustained patient loyalty. These findings are consistent with previous Chinese empirical evidence, where E-health literacy enhances patients’ health behaviors, improves doctor-patient relationships, and increases service satisfaction, laying a critical foundation for establishing high-quality relationships and maintaining long-term patient loyalty (12, 35, 40). Against the backdrop of rapid digitalization in county medical systems, this study further confirms that improving inpatients’ E-health literacy is a key pathway to strengthening their loyalty.

The study confirmed that value co-creation acts as a mediator between E-health literacy and patient loyalty among inpatients in county-level public hospitals. E-health literacy not only directly improves patient loyalty, but also indirectly enhances loyalty by promoting value co-creation behaviors during the healthcare service process. Specifically, inpatients with higher E-health literacy are better equipped to access and understand health information, proactively engage with digital tools in personalized diagnosis and treatment, share health data, and participate in mutual support communities. This transforms them from passive recipients of services to co-creators of the healthcare experience (24). Such active participation not only enhances patients’ sense of belonging and engagement, but also drives optimization of hospital workflows and improvement of service quality, ultimately increasing patient loyalty.

Perceived social support plays a significant moderating role in the relationship between E-health literacy and patient loyalty among inpatients in county-level public hospitals. Contrary to previous findings, this study found that as the level of perceived social support increases, the direct effect of E-health literacy on patient loyalty weakens. This phenomenon can be interpreted within the framework of Resource Substitution Theory (31), which suggests that when patients receive adequate external social support, these resources and trust buffers partially substitute for the direct effect of E-health literacy on loyalty. As a result, patient loyalty is no longer solely dependent on individual levels of E-health literacy, but is influenced by the combined effects of their social networks and available resources. Against the backdrop of a family-centered medical decision-making culture in China, this substitution effect may be particularly pronounced (41). Since patients tend to trust and follow health advice from family members rather than actively using digital tools to search for health information, the independent effect of e-health literacy is correspondingly attenuated. This finding further corroborates the buffering role of social support in the adoption of digital health services (42) and highlights the moderating significance of socio-environmental factors in the translation of e-health literacy into health behaviors and service loyalty.

Overall, this study has constructed and empirically validated the mediated moderation mechanism among E-health literacy, value co-creation, and patient loyalty in county-level inpatient populations in China, as well as the moderating role of perceived social support in the process by which E-health literacy fosters patient loyalty. These findings also carry targeted practical implications for multiple stakeholders. Specifically, for county hospitals, stratified and contextualized digital health education should be prioritized to enhance patients’ E-health literacy, enabling patients with varying levels of digital competence to effectively access online medical services and participate in health management (10). For medical IT organizations, improving the functionality and usability of online platforms can reinforce patient engagement, promote value co-creation behaviors, and ultimately enhance patient experience and loyalty (43, 44). For public health agencies, the moderating role of perceived social support indicates that integrating family- and community-level resources into digital health promotion strategies can strengthen intervention effectiveness. These implications provide actionable insights for optimizing service delivery and patient relationship management in resource-constrained settings.

Despite its contributions, this study has several limitations. First, the study population was restricted to county-level public hospitals in Shandong Province. Given the considerable regional disparities across China in terms of digital infrastructure, Internet penetration, and patient healthcare-seeking behaviors, the applicability of our findings to other regions or healthcare institutions at different tiers warrants further investigation. Second, as a cross-sectional study, it can only identify correlations among variables rather than establish causal relationships. Finally, data collection relied on self-reported questionnaires, which may introduce information bias.

In view of the above limitations, future studies should include patients from diverse geographic regions and healthcare institution tiers to enhance the generalizability of the findings. To establish causal relationships and temporal ordering, researchers could employ multi-wave follow-up surveys or quasi-experimental designs to track dynamic changes in the key variables over time. Similarly, to reduce biases associated with self-report measures, future research should incorporate objective behavioral indicators, such as patient portal usage logs or electronic health record data. From a broader perspective, subsequent investigations could further extend the theoretical framework from organizational and public health perspectives by examining how hospital operational models, medical platform service mechanisms, regional health policies, and other relevant factors jointly affect patient loyalty.

## 5 Conclusion

By employing a moderated mediation model, this study systematically explored the mechanism through which E-health literacy influences patient loyalty among inpatients in county-level public hospitals. The results indicate that E-health literacy can not only directly enhance patient loyalty but also indirectly do so by facilitating value co-creation behaviors. In addition, perceived social support plays a significant moderating role in the relationship between E-health literacy and patient loyalty. These findings expand the theoretical framework linking E-health literacy and patient loyalty and provide empirical evidence and strategic references for county-level public hospitals to optimize service models, improve patient engagement, and enhance loyalty in the context of digital health transformation.

## Data Availability

The raw data supporting the conclusions of this article will be made available by the authors, without undue reservation.
